# Implementing routine use of self-removed ureteric stents on extraction strings: prospective patient-reported outcome measures and complications

**DOI:** 10.1007/s00345-023-04653-z

**Published:** 2023-10-16

**Authors:** Jianliang Liu, Thomas P. Cundy, Natalie Parker, Mark Lloyd, Jonathan Cho, Rick L. Catterwell

**Affiliations:** 1 Department of Urology, The Queen Elizabeth Hospital, Woodville, SA Australia; 2 Discipline of Surgery, University of Adelaide, Adelaide, SA Australia

**Keywords:** Ureterolithiasis, Lithotripsy, Ureteric stent, Ureteral stent, Extraction string

## Abstract

**Purpose:**

Ureteric stents placed after ureteroscopy typically require cystoscopy for removal. Stent extraction strings allow the option of patient self-removal. This facilitates shorter stent dwell time, and cost-savings. Concerns regarding safety and limited evidence regarding patient acceptability are speculated reasons for infrequent clinical use of extraction strings. This study investigates our recent experience using routine self-removal of stents on extraction strings to provide evidence to address these concerns.

**Methods:**

In February 2020, our hospital adopted a policy for self-removal of stents on extraction strings to be routine following ureteroscopy. This was influenced by motivation to improve service capacity for diagnostic flexible cystoscopy, hospital avoidance during the pandemic, perceived improvement for the patient experience, and cost-saving. Prospective clinical and patient-reported outcome data were collected and evaluated.

**Results:**

There were 168 patients who had stents on extraction strings. Mean stent dwell time was 5.2 ± 1.8 days. Primary ureteroscopy was performed in 40.5%, and 59.5% had procedures using an access sheath. Self-removal at home was successful for 79% of patients. Stent dislodgement rate was 3.0% (5/168) and retained stents due to string detachment occurred in 1.8% (3/168). Almost all indicated they *“would remove the stent on string again”* (90%, 128/142) and the majority reported stent removal as *“very easy”* (61%, 87/142). Cost modelling estimates a total saving of AUD $148,869 per annum for routine use of extraction strings at our hospital.

**Conclusion:**

Our experience reflects that stent extraction strings may be used routinely with acceptable low complication rates, favourable patient experiences and associated cost savings.

## Introduction

Insertion of an indwelling ureteric stent after ureteroscopy is common, with estimates of use in 67–81% of these procedures [1–3]. This practice continues despite randomised controlled trials and guidelines indicating routine post-operative stenting is not necessary after uncomplicated procedures and might be associated with both higher morbidity and cost [4].The perceived benefits of ureteric stents after ureteroscopy are primarily to aid post-operative urinary drainage by preventing transient ureteric obstruction related to oedema, small stone fragments or clot [3].

The concept of an externalised stent extraction string was first introduced by Siegel et al*.* in 1986 who modified their stents by hand-threading a nylon suture through a distal stent fenestration [5]. Modern stents are now typically manufactured with this design using a long-looped fine extraction string. This allows the option of a string to be left externalised from the urethral meatus once the distal end of stent is deployed within the bladder. The extraction string allows patients to self-remove their stent at home and avoid an invasive cystoscopy procedure to achieve the same result. In addition to benefits of patient convenience, this also facilitates shorter stent dwell time with reduction in stent-related morbidity and cost [3, 6–8]. In countries like Australia where one-third of the population resides in rural and remote areas, it is an attractive option for patients to remove stents locally without need to travel long distances to a metropolitan hospital.

Survey data indicate that extraction strings are only used by approximately one-third of urologists, and < 10% allow patients to self-remove their stent [1, 8]. Reasons for infrequent use of stents with extraction strings remain unclear but are speculated to be contributed by concerns regarding lack of supportive evidence, patient acceptability, and risks for both stent dislodgement and string breakage causing stent retention [7, 8]. This study investigates our recent experience using routine patient self-removal of stents on extraction strings to provide evidence to inform and address these concerns.

## Materials and methods

In February 2020, our hospital adopted a policy for stents on extraction strings to be routine following retrograde intrarenal surgery (ureteroscopy, pyeloscopy) or percutaneous nephrolithotomy. Prior to this time, routine clinical care involved an indwelling ureteric stent that required subsequent cystoscopy for stent removal. Motivating factors for this change in clinical practice included strategy to improve service capacity to meet demand for diagnostic flexible cystoscopy procedures, hospital avoidance during the COVID-19 pandemic, perceived improvement for the patient experience, and cost-saving. Prospective clinical and patient-reported outcome data were collected to assess the outcome of this change.

There was a variety of stent size and type that included 4.8Fr or 6Fr JJ variable length stents, and both Cook (Cook Group, Indiana, United States) or Boston Scientific (Boston Scientific, Massachusetts, United States) products. All stents were variable length (also referred to as “multi-length”). Ureteric stent on strings were used as default for uncomplicated ureteroscopy, pyeloscopy, and percutaneous nephrolithotomy. Situations when indwelling stents without extraction string were considered more appropriate included iatrogenic ureteric trauma, solitary kidneys, transplant kidneys, requirement for more prolonged insertion time or questionable residual stone burden. Extraction string techniques varied between surgeons, ranging from a free-hanging tailored short string, to affixing looped string to the medial thigh (females) or penile shaft (males) with a transparent adhesive dressing with allowance for redundant length exiting the urethral meatus to accommodate mobility and erections.

Standard of care involved pre- and post-operative counselling regarding stent self-removal with a written patient information sheet provided. Patients were advised to self-remove their stent between 5 and 7 days following surgery. Discharge medications were limited to simple analgesia (paracetamol and ibuprofen), along with advice to generously hydrate. Alpha blockers, anticholinergics or opiates are not routinely provided on discharge.

A follow up telephone call was scheduled 2 weeks following surgery to confirm successful stent removal and collect a standardised patient-reported outcome measure questionnaire. For the non-dichotomous questionnaire items, patients were asked to grade their responses using a 10-point Likert scale between the extremes outlined above. Patients were also asked if they self-removed the stent or, if not, then who helped them to remove the stent. The questionnaire included these five questions;

- *“were the instructions for stent removal clear?”* (yes or no)
- *“how anxious were you about the stent removal?”* (1 to 10; “1” = not concerned at all, “10” = terrified)
- *“how difficult was it to remove the stent?”* (1 to 10, “1” = very easy, “10” = extremely complicated or unable to do it)
- *“how painful was the stent removal?”* (1 to 10; “1” = zero pain, “10” = excruciating)
- *“would you remove the stent on string again?”* (yes or no).

All scheduled follow-up patient telephone calls were conducted by specialist urology nurses. These data were contemporaneously recorded in the hospital electronic medical record and in a separate prospective database. Our study population were consecutive patients who had post-operative stents on extraction strings registered in the database. Clinical outcome data were collected from the electronic medical record, specifically stent-related complications. The study period was 32 months from February 2020 to September 2022. Cost modelling was based on our hospital episode costing of AUD $2363 for a flexible cystoscopy and stent removal procedure under local anaesthesia in the day surgery unit.

Data analysis was performed with intention-to-treat principle (e.g., inadvertently retained stents not excluded from analyses). The Chi-square test was used to compare complication rates between gender sub-groups. The Kruskall–Wallis test was used to compare Likert scale patient-reported outcome measures between gender sub-groups. Associations between patient-reported anxiety levels with stent removal and both stent removal difficulty and pain were evaluated using the Pearson correlation test. Statistical analysis was undertaken using SPSS version 28.0 (IBM Corp, New York).

## Results

There were 168 patients who had post-operative stents on extraction strings during the study period. The mean patient age (± SD) was 54.4 (± 17.5) years. Gender distribution was 1.27:1 for male:female patients (94 men, 74 women). Mean stent dwell time was 5.2 ± 1.8 days. Primary ureteroscopy or pyeloscopy was performed in 40.5% (68/168), and most of our study cohort had procedures involving use of a flexible ureteral access sheath (59.5%, 100/168). There were 3 percutaneous nephrolithotomy procedures that involved stents on extraction strings. Patient demographic information and indications for ureteric stents are summarised in Table 1.

**Table 1:** Demographics and indications of ureteric stent on string for included patients

	Ureteric stent on string (*n* = 168)
Male (*n* (%))	94 (56%)
Female (*n* (%))	74 (44%)
Mean stent dwell time (days ± standard deviation)	5.2 ± 1.8
Indication	
Primary ureteroscopy/pyeloscopy	68 (40.5%)
Staged ureteroscopy/pyeloscopy	97 (57.7%)
Percutaneous nephrolithotomy	3 (1.8%)

Despite intention and education for self-removal, stents were removed by someone else in 21.4% of occasions (36/168). Most of these stents were removed by clinicians (30/36) across a variety of healthcare settings that included the primary care general practice clinic (*n* = 10), hospital outpatient urology department (*n* = 9), emergency department (*n* = 8), and hospital inpatient ward (*n* = 3). Reasons reported for patients requesting family or friends to remove were either anxiety, mobility issues, or limited manual dexterity.

### Complications

The overall rate of stent dislodgement was 3.0% (5/168). There was no difference in dislodgement rates between men (2/6) and women (3/6; *p* = 0.85). All dislodgements involved accidental traction on the string resulting in extruded stent visible at the meatus. Three stent dislodgements occurred within 12 h of hospital discharge. One extruded stent was reinserted by manual re-positioning using a Foley catheter (female patient). All remaining dislodgement events were managed with complete removal of the stent after extrusion. No patients were required to return to the operating theatre for stent reinsertion.

Retained stents due to either string detachment or string breakage occurred in 3 patients (1.8%; 2 males, 1 female). Each retained stent was subsequently removed by flexible cystoscopy within 6 weeks following insertion. One patient unsuccessfully attempted self-removal on the instructed day and informed our specialist nurse only at the time of the scheduled follow-up phone call. Another patient informed our specialist nurse of “successful” self-removal at the scheduled follow-up phone call, but had a retained stent confirmed on a planned CT scan 5 weeks after surgery. It was later confirmed that this patient, with limited English-speaking fluency, mistook the detached string as the stent. The third patient unexpectedly observed the detached string in her underwear and alerted our clinical team.

### Patient-reported outcome measures

The completed questionnaire response rate was 83% (140/168). When contacted within the first 2 weeks after surgery, 90% of respondents (128/142) indicated they *“would remove the stent on string again”*. Almost all respondents reported that the *“instructions for stent self-removal were clear”* (98%, 142/145).

**Fig. 1:**
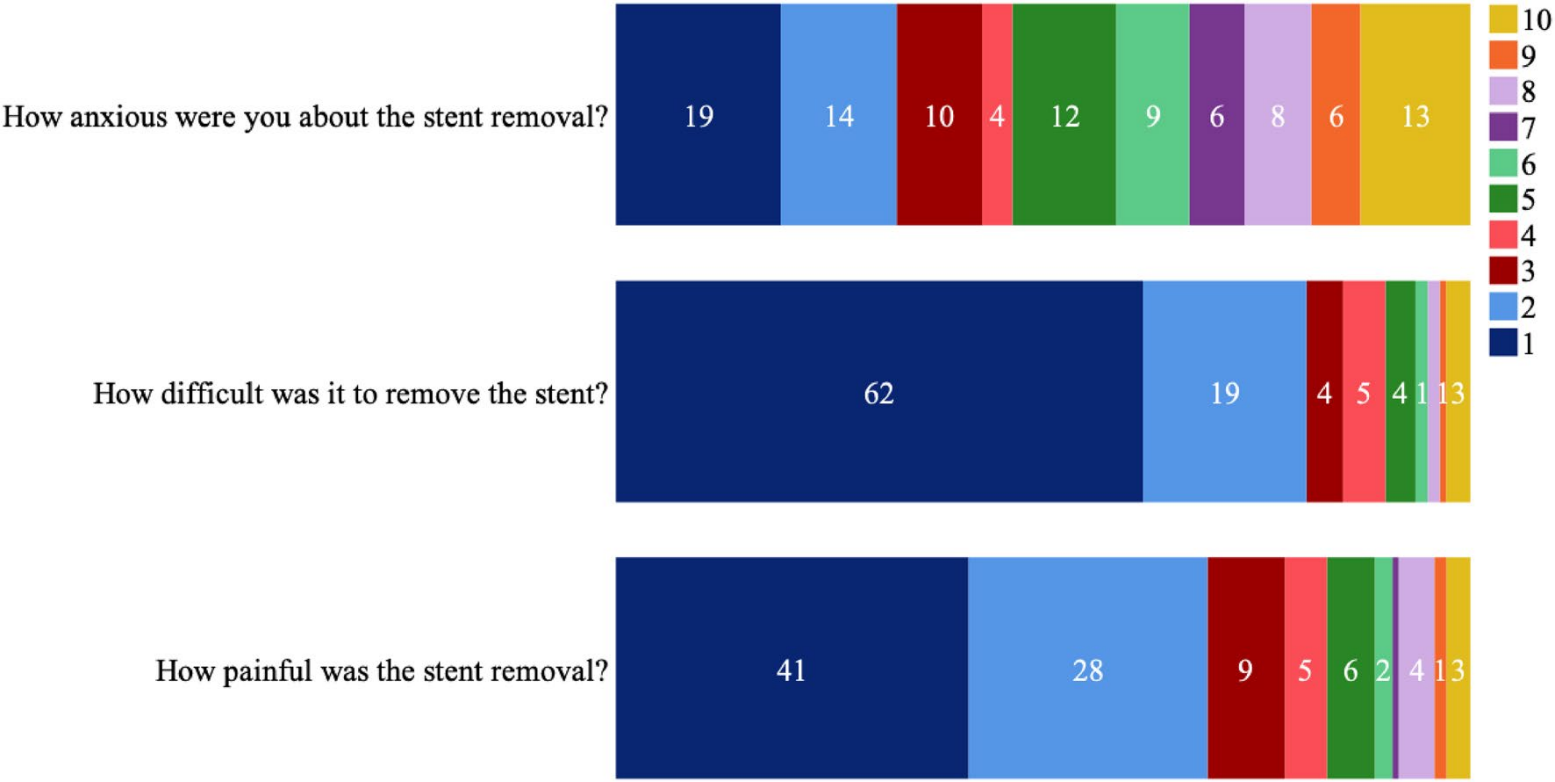
Distribution of 10-point Likert scale responses to patient experiences of ureteric stent self-removal using an extraction string (%)

Likert scale question response data are summarised in Fig. 1. Median aggregated 10-point Likert scale score for degree of difficulty of stent removal was 1 *“very easy”* (IQR 1). Stent removal was reported as *“very easy”* in 61% (87/142) of respondents when asked *“how difficult was it to remove the stent?”.* There was a mixed response to the question *“how anxious were you about the stent removal?”* with median aggregated Likert scale score of 5 (IQR 6). There was no difference in reported level of anxiety scores between male and female patients (*p* = 0.73). Median aggregated 10-point Likert scale score for degree of reported pain with stent removal was 2 (IQR 2). There was no statistically significant difference between male and female patients for degree of reported pain (*p* = 0.072).

Patients who reported higher levels of anxiety about stent removal were significantly more likely to report higher levels of pain with stent removal (*r* = 0.30; *p* < 0.001), and how difficult the stent removal was (*r* = 0.41; *p* < 0.001).

### Cost

Over the study period, there was an average of 5.2 self-removal of stent events per month. Cost modelling based on this frequency of stent self-removal estimates a total cost saving of AUD $148,869 per annum.

## Discussion

Literature reporting outcomes for use of ureteric stents on extraction strings is surprisingly limited given the almost ubiquitous use of stents in endourology. A recent systematic review by Oliver et al*.* identified < 500 published cases of stent on extraction strings use [8]. Our prospective series of consecutive patients contributes data on the use of these stents more broadly on a *routine* basis in clinical practice. This contrasts with the existing literature that focuses on use in more selected settings. Overall, our findings indicate stent self-removal using extraction strings to have an acceptable low complication rate, favourable patient experience and healthcare spending benefit.

The stent dislodgement rate of 3.0% we experienced was lower than the pooled rate of 9.9% reported by Oliver et al*.* [8]. We did not observe difference in dislodgement rates between male and female patients unlike previous studies that have identified significantly higher rates of dislodgement in females up to 24.4% compared to lower rates of 5.3% in males [9]. Reasons for these lower dislodgement rates are unclear. Various techniques of string tailoring or securement were used in our patient cohort as described above, including the free-hanging shortened string technique used in the randomised controlled reported by Barnes et al*.* [6]. Anecdotally, we have learned lessons through patient feedback during the study period, notably that male patients experience painful involuntary erections when a circumferential dressing is used to secure a loose redundant length of string to their penile shaft. Similarly, a string secured to the medial thigh of a female patient with a simple transparent adhesive dressing is often reported to pull against an underwear seam and be uncomfortable. For these reasons, we have erred towards the tailored free-hanging string technique previously reported by others [6, 10]. The security of this “unsecured” extraction string technique compared to other string management options is an opportunity for further study examining means to minimise dislodgement.

String breakage with resultant stent retention occurred in 1.8% of our study cohort. This complication is a rare event in other series [6]. These events highlight that although the extraction string should diminish the risk of a *“forgotten”* stent maintenance of a contemporaneous local stent registry or similar audit process remains important.

The intention for all stents was that patients would self-remove their stents in their own home. Our finding that 21% of patients required someone else to remove their stent was higher than expected. Successful self-removal rates are reported as high as 97% in a clinical trial setting [6] and 88% in a systematic review pooled analysis [6]. We do not consider having someone else remove a stent to be a failure of the concept of a stent on extraction string because a cystoscopy procedure is still successfully avoided, and timely removal is still achieved within the desired period. Given that most of the non-self-removal events involved healthcare professionals across a range of clinical settings, this does have a hidden financial cost and seems detrimental to the desired convenience gain for patients.

An alternative to a stent on extraction string is an open-ended ureteric catheter that is externalised into a urinary catheter for removal usually the day after surgery. The principles of this serve a similar role to an extraction string, however, it does require the patient to stay in hospital overnight. Elective ureteroscopy at our hospital is usually performed as a day procedure case. Magnet-assisted stent removal has been recently introduced as another consideration to enable stent removal without cystoscopy. This concept consists of a specific stent product with a magnet at the distal end that can then be retrieved by insertion of a magnet retrieval device such as a magnetised catheter [11]. Although the magnet design principle is innovative, this option still necessitates an invasive bedside procedure for stent removal that we suggest is less appealing than an extraction string removal.

In our hospital, discussion regarding the option of stent self-removal often occurs on the day of surgery during the first interaction between the surgeon and patient. This is not ideal but a reality of a high-volume university teaching hospital. Patients who strongly object or have an important clinical indication for indwelling post-operative stent are not left with an extraction string for self-removal. Otherwise, the routine is a plan for stent self-removal. With this approach during our study period patient-reported experiences were overall highly positive, but this was not universal. Detailed pre-operative discussion and providing written information instructions on discharge are key contributors to a positive patient experience, but we believe there is scope to provide more nuanced patient selection. Future research is needed to identify patients who are best-suited candidates for a successful and positive stent self-removal experience. Conversely, further decision support data would be helpful to guide patient selection of those who would be poorly suited for stent self-removal. We have not found extremes of age (range 19–93 years) or gender to be factors that influence complication rates or patient experience for stent self-removal. Patient-reported higher anxiety about stent removal was strongly correlated with an experience of difficult and painful stent removal, so this factor warrants greater consideration in patient selection.

We appreciate limitations with our prospective study design at a single hospital site. These include a lack of randomization for comparative assessment of the stent on extraction string option, and that our results might not be as generalisable as a multi-hospital study design. The clinical outcomes reported in this manuscript were focused on short-term follow-up as any complications arising specifically from use of self-removal by extraction string were expected to manifest in the early post-operative period. However, longer-term outcomes of stents on extraction strings have not been investigated in this study and remain under-reported in the literature.

## Conclusion

Our experience indicates that self-removal of stents on extraction strings may be used routinely with acceptable low complication rates, favourable patient experiences and associated cost savings. Almost one-quarter of patients required another person to remove their stent. Future research to better predict this patient group in advance would improve the overall patient experience with extraction strings.

## Data Availability

No identifiable data were used for this study. All data have been managed appropriately under the Australian code of the Responsible Conduct of Research.
